# Drastic Improvement in Adhesion Property of Polytetrafluoroethylene (PTFE) via Heat-Assisted Plasma Treatment Using a Heater

**DOI:** 10.1038/s41598-017-09901-y

**Published:** 2017-08-25

**Authors:** Yuji Ohkubo, Kento Ishihara, Masafumi Shibahara, Asahiro Nagatani, Koji Honda, Katsuyoshi Endo, Kazuya Yamamura

**Affiliations:** 1 Graduate School of Engineering, Osaka University, 2-1 Yamadaoka, Suita, Osaka 565-0871 Japan; 2 Hyogo Prefectural Institute of Technology, 3-1-12 Yukihiracho, Kobe, Hyogo 654-0037 Japan

## Abstract

The heating effect on the adhesion property of plasma-treated polytetrafluoroethylene (PTFE) was examined. For this purpose, a PTFE sheet was plasma-treated at atmospheric pressure while heating using a halogen heater. When plasma-treated at 8.3 W/cm^2^ without using the heater (Low-P), the surface temperature of Low-P was about 95 °C. In contrast, when plasma-treated at 8.3 W/cm^2^ while using the heater (Low-P+Heater), the surface temperature of Low-P+Heater was controlled to about 260 °C. Thermal compression of the plasma-treated PTFE with or without heating and isobutylene−isoprene rubber (IIR) was performed, and the adhesion strength of the IIR/PTFE assembly was measured via the T-peel test. The adhesion strengths of Low-P and Low-P+Heater were 0.12 and 2.3 N/mm, respectively. Cohesion failure of IIR occurred during the T-peel test because of its extremely high adhesion property. The surfaces of the plasma-treated PTFE with or without heating were investigated by the measurements of electron spin resonance, X-ray photoelectron spectroscopy, nanoindentation, scanning electron microscopy, and scanning probe microscopy. These results indicated that heating during plasma treatment promotes the etching of the weak boundary layer (WBL) of PTFE, resulting in a sharp increase in the adhesion property of PTFE.

## Introduction

Fluoropolymers have several excellent properties such as high hydrophobicity, high oleophobicity, high chemical resistance, high antifouling property, high sliding property, high thermal resistance, high weather resistance, low relative permittivity, and low dielectric loss tangent. They are, however, difficult to adhere to other types of materials because of their low surface energy^1, 2^. Of all resins, polytetrafluoroethylene (PTFE) is the most difficult to adhere. Therefore, corrosive materials such as sodium–naphthalene and sodium–ammonium complex solutions have been used to improve its adhesion property to other materials^3, 4^. However, the solutions have a bad smell and dramatically impact on humans and the environment. In addition, sodium residues remain on PTFE. Therefore, an alternative method without using sodium-containing solutions has been long needed. As an alternative method without using sodium-containing solutions, surface treatment based on ion irradiation has been reported by Yumoto’s group^5–10^. In one of their reports^9^, the adhesion strength between PTFE and epoxy adhesives increased to 1.5 N/mm upon N_2_ ion irradiation. Although the adhesion property significantly improves, the ion irradiation is performed under low pressure (1.3 × 10^−2^ Pa). This indicates that the ion irradiation absolutely requires a vacuum evacuation system and is difficult to continuously treat the PTFE surface and to treat the large area of PTFE surface. In contrast, plasma treatment is able to be performed under atmospheric pressure, which means that plasma treatment has possibility to realize a high-throughput surface treatment of PTFE. Therefore, plasma treatment has a bigger advantage for practical use than ion irradiation. There are many reports on plasma treatment for PTFE using single gases such as Ar^11–14^, CF_4_^11^, CO_2_^15^, H_2_^11, 16^, H_2_O^15^, He^11^, N_2_^11–13, 16, 17^, Ne^11^, NH_3_^12, 13^, and O_2_^11–13, 17, 18^, and mixed gases such as Ar + O_2_^14^, He + H_2_O^19^, He + O_2_^20^, and N_2_ + H_2_^16^. The relationship among plasma treatment conditions, chemical composition, morphology, and wettability have been studied and reported in detail. However, few reports deal with the adhesion property of plasma-treated PTFE. Therefore, our research group focused on the improvement of the adhesion property of PTFE and started to develop an alternative method for improving it using plasma treatment. We found and reported that heat-assisted plasma treatment drastically improved the adhesion property of PTFE^21^. Although no adhesive agents were present, the heat-assisted plasma-treated PTFE strongly adhered to unvulcanized isobutylene−isoprene rubber (IIR) and cohesion failure of the rubber occurred during the T-peel test instead of interfacial peeling. In the previous report, we suggested that heat-assisted plasma treatment induces not only surface modification, but also surface hardening through the formation of C–C cross-links and/or etching of weak boundary layers (WBL). In other words, peroxide radicals (C–O–O^•^) and active functional groups containing oxygen (O–C=O, C=O, C–O) existing on the steady ground layer cause the extremely high adhesion of the IIR/PTFE assembly. However, in the previous report, the surface temperature of PTFE was controlled by applying radio-frequency (RF) power for plasma generation. Although the adhesion property increased upon increasing the applied RF power density and the surface temperature, the radical density ratio also increased. In summary, the adhesion property was improved by both the heating effect and the increase in radical amount. For clarifying the actual heating effect on adhesion property, it is needed to divide heat-assisted plasma treatment into two systems: the plasma irradiation part and heating part using a heater. In addition, if the heating using a heater during plasma treatment also give a high adhesion to PTFE, it would have a big advantage for practical use. Adding the heater to an existing plasma treatment equipment is easier for increasing the surface temperature of PTFE than control of applied RF power density. Because the applied RF power density is often limited in the case of plasma treatment for large area. Therefore, confirming the actual heat effect on adhesion property of PTFE using a heater is significant important. In this study, we added a heater to the plasma-treatment equipment then compared the two kinds of heat-assisted plasma treatments: heating with increasing applied RF power density and heating using a heater, in their ability to modify the PTFE surface.

## Results

### Heating effect on adhesion strength

**Figure 1:**
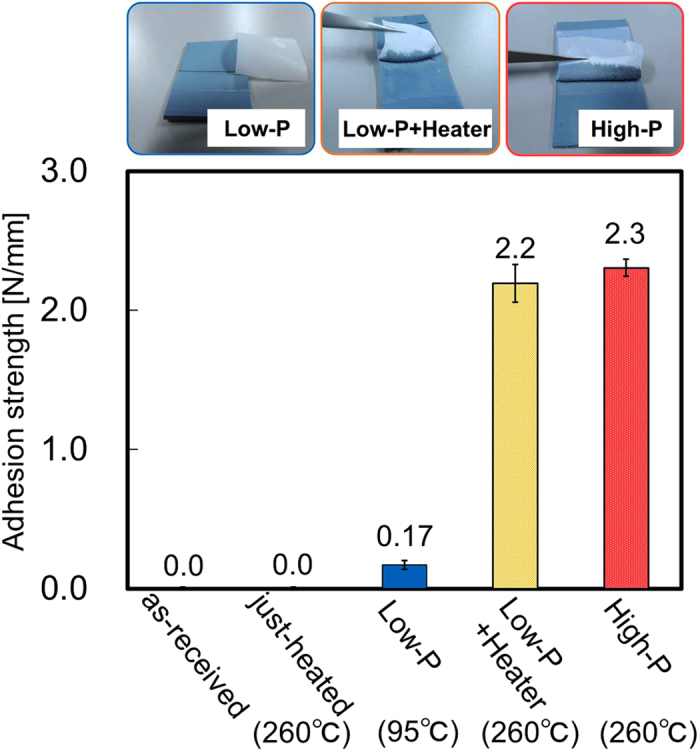
Photographs of IIR/PTFE assembly sample after the T-peel test and the adhesion strengths (n = 3).

**Figure 2:**
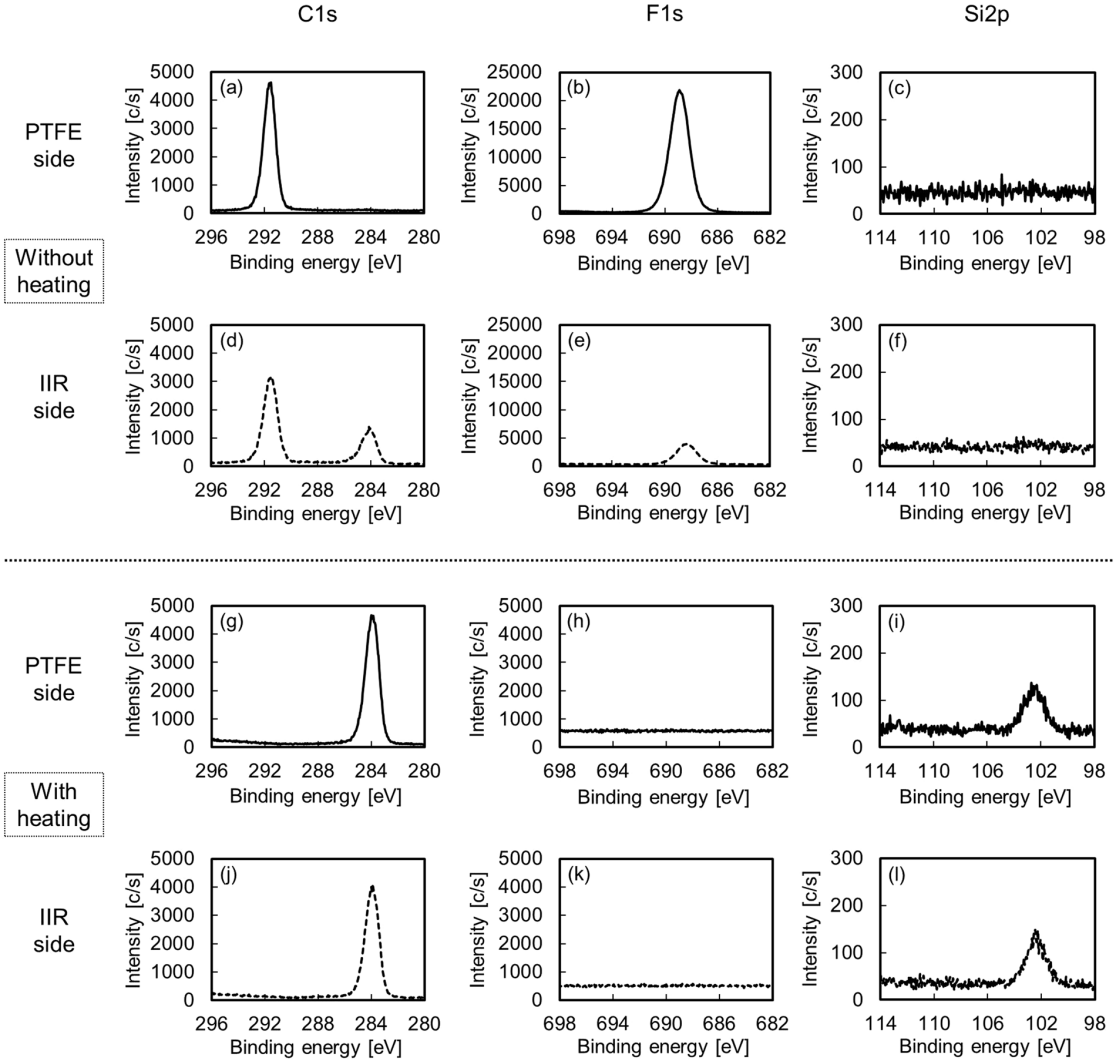
XPS spectra (take-off angle of 45°) of the peeled surfaces with or without heating. (**a**) C1s-XPS of PTFE side without heating, (**b**) F1s-XPS of PTFE side without heating, (**c**) Si2p-XPS of PTFE side without heating, (**d**) C1s-XPS of IIR side without heating, (**e**) F1s-XPS of IIR side without heating, (**f**) Si2p-XPS of IIR side without heating, (**g**) C1s-XPS of PTFE side with heating, (**h**) F1s-XPS of PTFE side with heating, (**i**) Si2p-XPS of PTFE side with heating, (**j**) C1s-XPS of IIR side with heating, (**k**) F1s-XPS of IIR side with heating, (**l**) Si2p-XPS of IIR side with heating.

We prepared five PTFE samples denoted as-received, just-heated, Low-P, Low-P+Heater, and High-P, as shown in Table 1. Figure 1 shows the photographs of the IIR/PTFE assembly sample after the T-peel test and the adhesion strengths of the PTFE samples. The adhesion strength of the Low-P PTFE sample was slightly higher than that of the as-received PTFE sample. The Low-P+Heater PTFE sample presented a drastically stronger adhesion to IIR than the Low-P PTFE sample. Its adhesion strength of the Low-P+Heater PTFE sample was more than 2.0 N/mm. The chemical compositions of the peeled surfaces with or without heating were examined by XPS measurements. Figure 2 shows the XPS spectra of the peeled surfaces with or without heating. In the case of without heating during plasma treatment, CF_2_ and F derived from PTFE were detected but Si derived from IIR was not detected on the peeled surfaces of both PTFE and IIR sides (Figs. 2a–2f). These results indicated that the peeling occurred at the PTFE surface layer but did not occur at the interface of PTFE and IIR. In the case of heating during plasma treatment, CF_2_ and F derived from PTFE were not detected at all but C-H and/or C-C and Si derived from IIR were detected on the peeled surfaces of both PTFE and IIR sides (Figs. 2g–2l). These results indicated that the peeling occurred at the rubber bulk layer. In other words, cohesion failure of rubber occurred for the heat-assisted plasma-treated PTFE samples: both Low-P+Heater and High-P samples. In contrast, the adhesion strength of just-heated PTFE at 260 °C was 0.0 N/mm. This result corresponds to the effect of heating during plasma treatment on the adhesion property of PTFE.

**Table 1:** PTFE sample IDs and experimental conditions.

Sample ID	Plasma	Heating
as-received	—	—
just-heated	—	○(260 °C)
Low-P	○(8.3 W/cm^2^)	—(95 °C)
Low-P+Heater	○(8.3 W/cm^2^)	○(260 °C)
High-P	○(21.7 W/cm^2^)	—(260 °C)

### Heating effect on chemical and physical properties of PTFE surface

**Figure 3:**
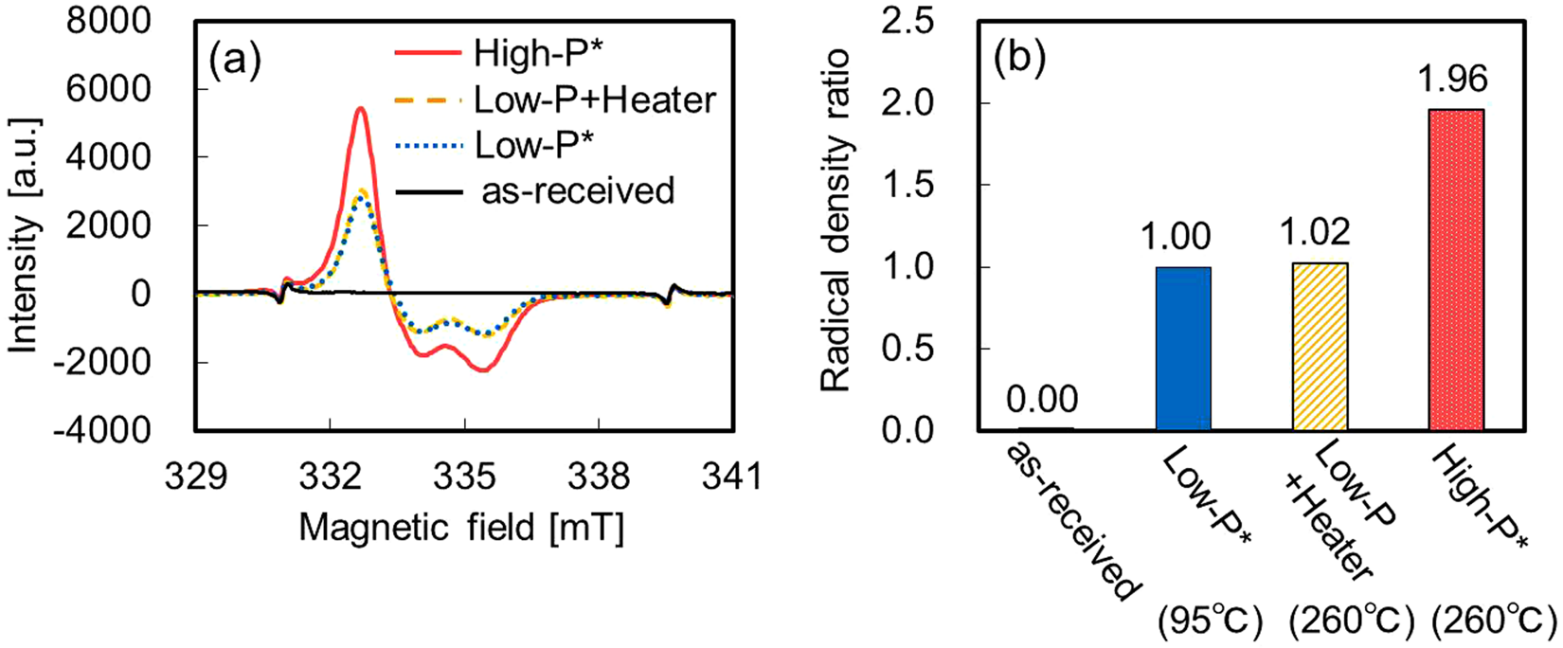
ESR data of the PTFE samples with or without plasma treatment and with or without heating. (**a**) ESR spectra and (**b**) radical density ratio. *The data of Low-P and High-P PTFE samples are the same as in our previous report^21^.

The heating effect on the PTFE surface was investigated to explain the sharp increase in adhesion strength. Figure 3a shows the ESR spectra of the PTFE samples with or without plasma treatment and with or without heating. Except for the ESR spectra of as-received PTFE, broad peaks indexed to the peroxide radical (C–O–O^•^) were observed between 332 and 337 mT in all ESR spectra. The ESR spectrum of just-heated PTFE had no peaks indexed to the peroxide radical (not shown here). It is known that peroxide radicals are formed through the reaction between the carbon radicals generated by defluorination of CF_2_ and the oxygen molecules in the atmosphere. We can discriminate two types of peroxide radicals, the alkyl-type (–CF_2_CFOO^•^CF_2_–) and the methylene-type radical (–CF_2_CF_2_CF_2_OO^•^)^22^ from the shape of the ESR spectra. A symmetric spectrum indicates that methylene-type peroxide radicals are present because of the free rotation of the peroxide radical. In contrast, an asymmetric spectrum indicates alkyl-type peroxide radicals because of the partially-restricted rotation of the peroxide radical. Consequently, the peroxide radicals in this study contain more alkyl-type than methylene-type radicals. This result indicates that the scission of the C–C main chain was less pronounced than that of the scission of C–F side chain, which means that the PTFE surface was only softly modified. Figure 3b shows the radical density ratios of the PTFE samples with or without plasma treatment and with or without heating. The radical density ratio of the High-P PTFE sample was about twice as high as that of the Low-P PTFE sample. This result indicates that the applied RF power density affects the radical formation. In contrast, the radical density ratio of the Low-P+Heater PTFE sample was almost similar to that of the Low-P PTFE sample. This result means that heating during plasma treatment does not affect the radical density ratio. In summary, the radical density ratio of the Low-P PTFE sample was in principal sufficiently high to adhere PTFE to other materials, but other factors were also present that prevented it from adhesion.

**Figure 4:**
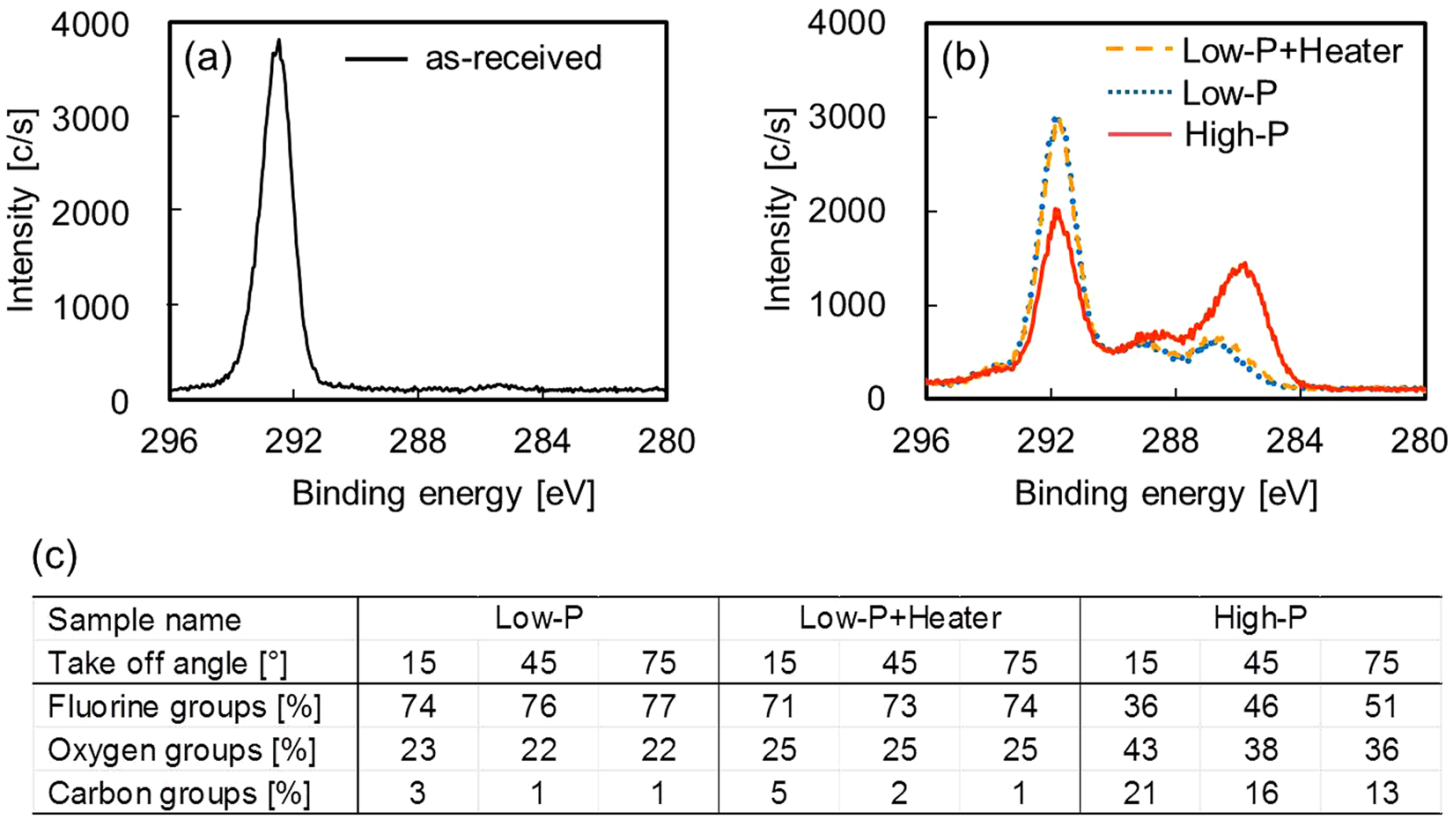
C1s-XPS spectra (take-off angle of 45°) of the PTFE samples with or without plasma treatment and with or without heating. (**a**) as-received, (**b**) plasma-treated, and (**c**) chemical components calculated from angular-dependent XPS spectra (S-1).

Chemical components of plasma-treated PTFE surface were investigated by angular-dependent XPS measurements. S-1 shows all the angular-dependent XPS spectra of Low-P, Low-P+Heater, and High-P and the results of the peak resolutions in detail. Figures 4a and b show the representative C1s-XPS spectra (take-off angle of 45°) of the PTFE samples with or without plasma treatment and with or without heating. Except for the XPS spectra of as-received PTFE and just-heated PTFE (not shown here), the intensity of the peak indexed to fluorine-containing functional groups (CF_3_, CF_2_, C–F) at ca. 292 eV decreased compared with that of the as-received PTFE upon plasma treatment. This result indicated that C–F bond scissions occurred on the PTFE surface upon plasma treatment. As shown in S-1, a small amount of CF_3_ was detected, which indicates that some carbon radicals react with fluorine atoms in plasma. The intensities of the peak indexed to oxygen-containing functional groups (O–C=O, C=O, C–O) increased relative to those of the as-received PTFE sample upon plasma treatment and surface exposure to atmospheric oxygen after plasma treatment. This result indicated that carbon radicals react with oxygen and moisture in the air. The intensities of the peaks attributed to main carbon groups (C–C, C–H, C=C) increased as compared to those of the as-received PTFE upon plasma treatment. This result suggested that carbon radicals react with other carbon radicals, which results in the formation of C–C cross-links. It was especially interesting that the shape of the C1s-XPS spectra of Low-P+Heater PTFE sample was similar to that of Low-P PTFE sample. Figure 4c shows the chemical components calculated from angular-dependent XPS spectra (S-1). The difference between Low-P and Low-P+Heater PTFE samples was within 3%. Taking into account the results of the T-peel test, different spectra were expected for the Low-P and Low-P+Heater PTFE samples. On the other hand, similar C1s-XPS spectrum shapes were expected for the Low-P+Heater and High-P PTFE samples. This result implies that the surface composition of Low-P PTFE sample was suitable to adhere PTFE to other materials, but other factors would decrease the adhesion strength of PTFE.

**Figure 5:**
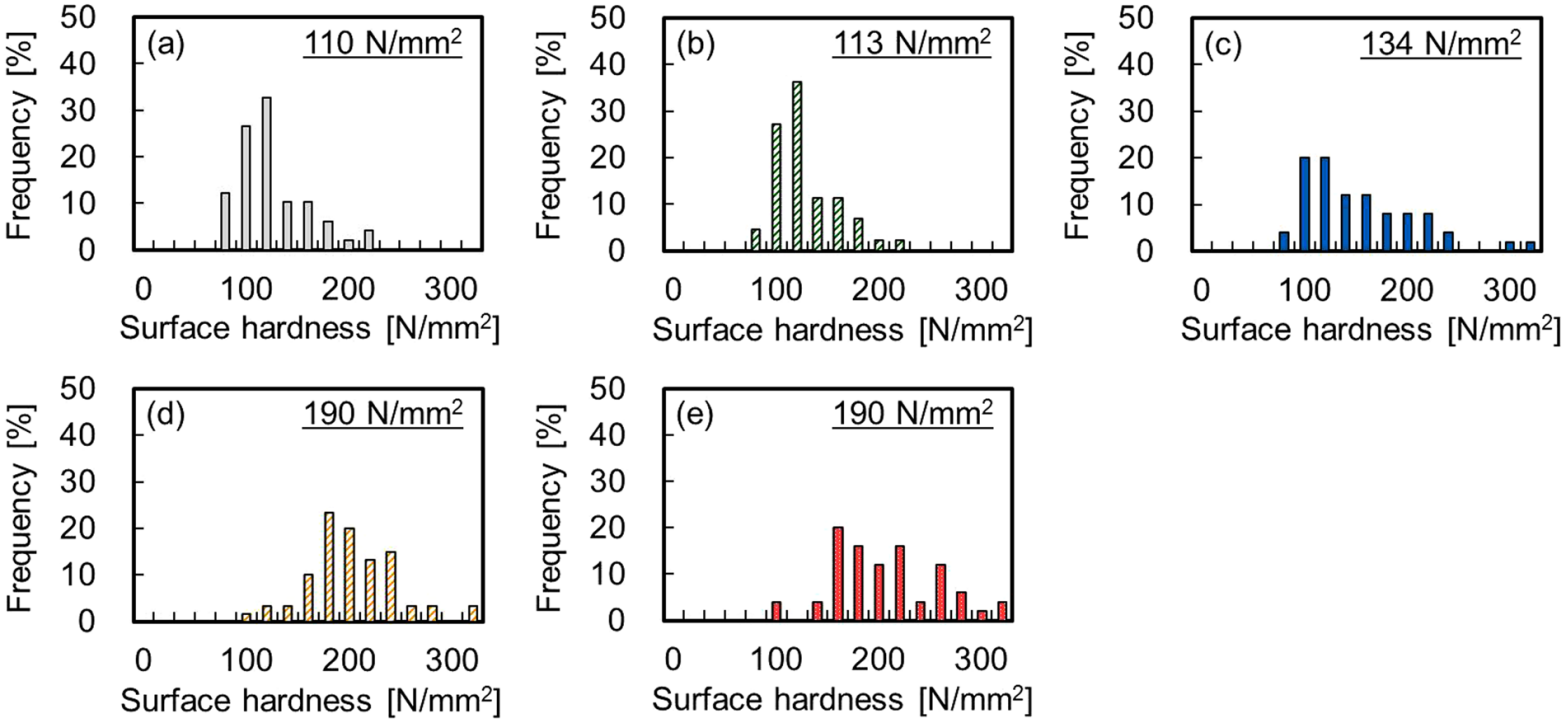
Surface hardness of the PTFE samples with or without plasma treatment and with or without heating. (**a**) as-received, (**b**) just-heated, (**c**) Low-P, (**d**) Low-P+Heater, and (**e**) High-P.

Figure 5 shows the average surface hardness of the PTFE samples with or without plasma treatment and with or without heating. The surface hardness of the Low-P+Heater PTFE sample (190 N/mm^2^) was higher than that of the Low-P PTFE sample (134 N/mm^2^) and approached that of the High-P PTFE sample (190 N/mm^2^). In contrast, the surface hardness of the just-heated PTFE was 113 N/mm^2^, which indicated that just heating had no effect on the increase in surface hardness. Furthermore, heating during plasma treatment further hardens the PTFE surface. This result indicated that an increase in surface hardness of the plasma-modified layer containing peroxide radicals and oxygen-containing functional groups was of critical importance for improving the adhesion property of PTFE. Two factors increase the surface hardness upon heat-assisted plasma treatment: (1) the promotion of etching reactions in the weak boundary layer (WBL) on the PTFE surface, (2) the promotion of the formation of C–C cross-links.

### Heating effect on PTFE surface topography

**Figure 6:**
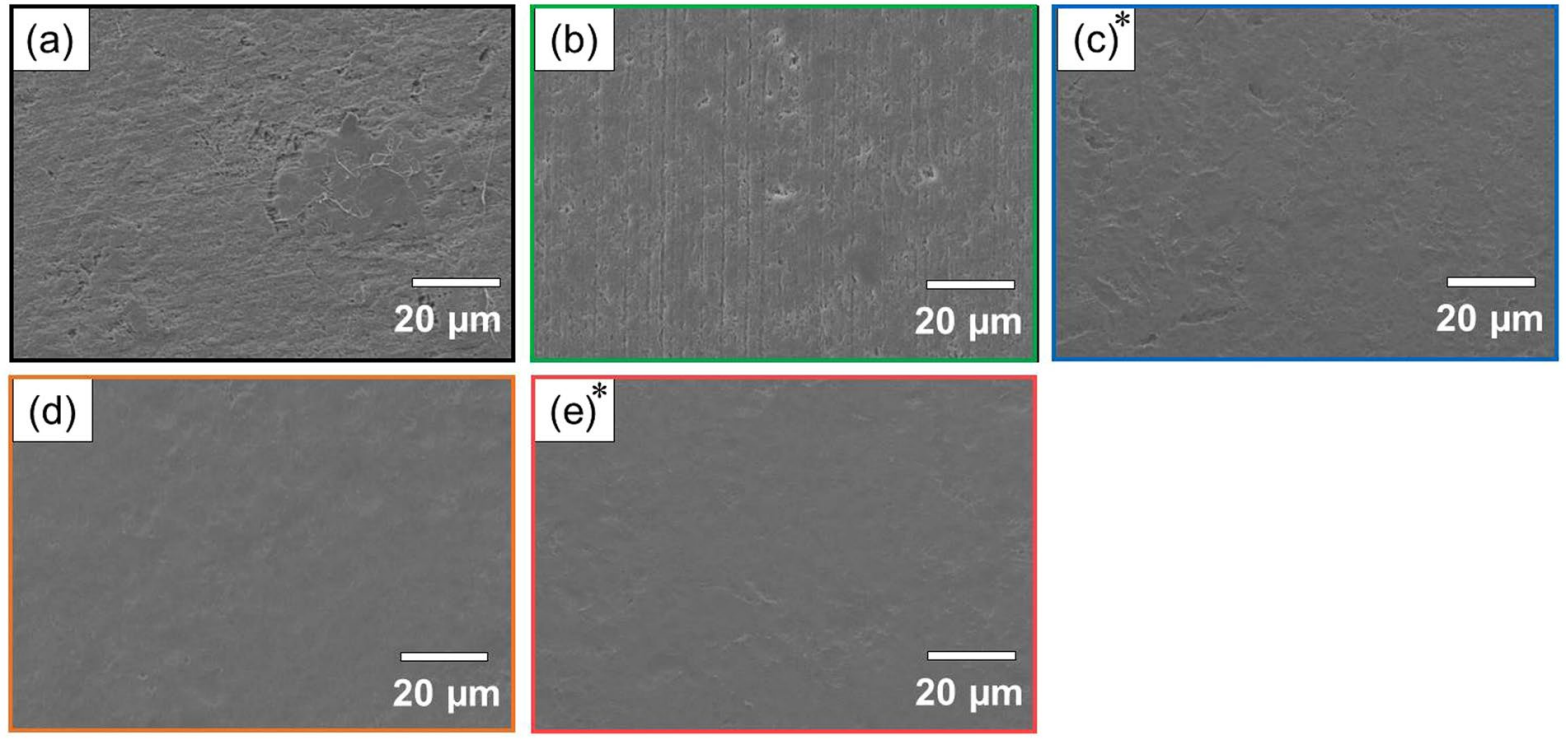
SEM images of the PTFE samples with or without plasma treatment and with or without heating. (**a**) as-received, (**b**) just-heated, (**c**) Low-P, (**d**) Low-P+Heater, and (**e**) High-P. *The data of Low-P and High-P PTFE samples are the same as in our previous report^26^.

Figure 6 shows the SEM images of the PTFE samples with or without plasma treatment and with or without heating. Although many cutting scratches and pits were observed on the as-received PTFE surface (Fig. 6a), they disappear upon plasma treatment (Figs. 6c–6e). In addition, the size of pits and the surface roughness further decreased upon heat-assisted plasma treatment (Figs. 6d and 6e). In contrast, many cutting scratches and pits remained on the just-heated PTFE surface (Fig. 6b). These results indicated that only heating had no effect on the surface morphology and simultaneous plasma treatment and heating is the most important procedure for changing the morphology.

**Figure 7:**
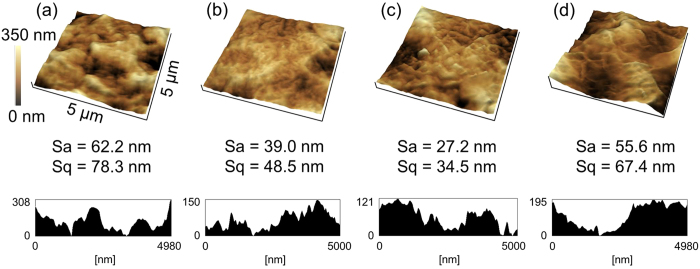
AFM images of the PTFE samples with or without plasma treatment and with or without heating. (**a**) as-received, (**b**) Low-P, (**c**) Low-P+Heater, and (**d**) High-P.

Figure 7 shows the AFM images of the PTFE samples with or without plasma treatment and with or without heating. The surface roughness Sa of the as-received PTFE was 62.2 nm. In contrast, the surface roughness Sa of Low-P, Low-P+Heater, and High-P samples were 39.0, 27.2, and 55.6, respectively. In summary, the surface roughness of plasma-treated PTFE samples decreased as compared to the as-received PTFE, but the difference of Sa values was not large. Sq values indicated similar behavior to Sa ones. These results were consistent with the previous reports^11, 12^. Although the Sa and Sq values did not drastically change upon plasma treatment, the surface topography certainly changed. The surface of the as-received PTFE had rounded irregularities (Fig. 7a). In contrast, the surface of the plasma-treated PTFE became less round and more angular in shape (Figs. 7b–7d). Especially, the formed protrusion on the plasma-treated PTFE surface with heating seemed to become finer and sharper than without heating. This result implies that an etching reaction was promoted by heating during plasma treatment. As shown in Fig. 6a, as-received PTFE has a WBL layer. Hubert *et al*. previously reported that the amorphous part was more likely to be etched by plasma treatment than crystalline part^20^. For the amorphous part, the molecular weight and hardness are predicted to be low, which results in weak interactions of CF_2_ chains with each other. The WBL was removed by the heat-assisted plasma treatment, which resulted in increased surface hardness.

## Discussion

**Figure 8:**
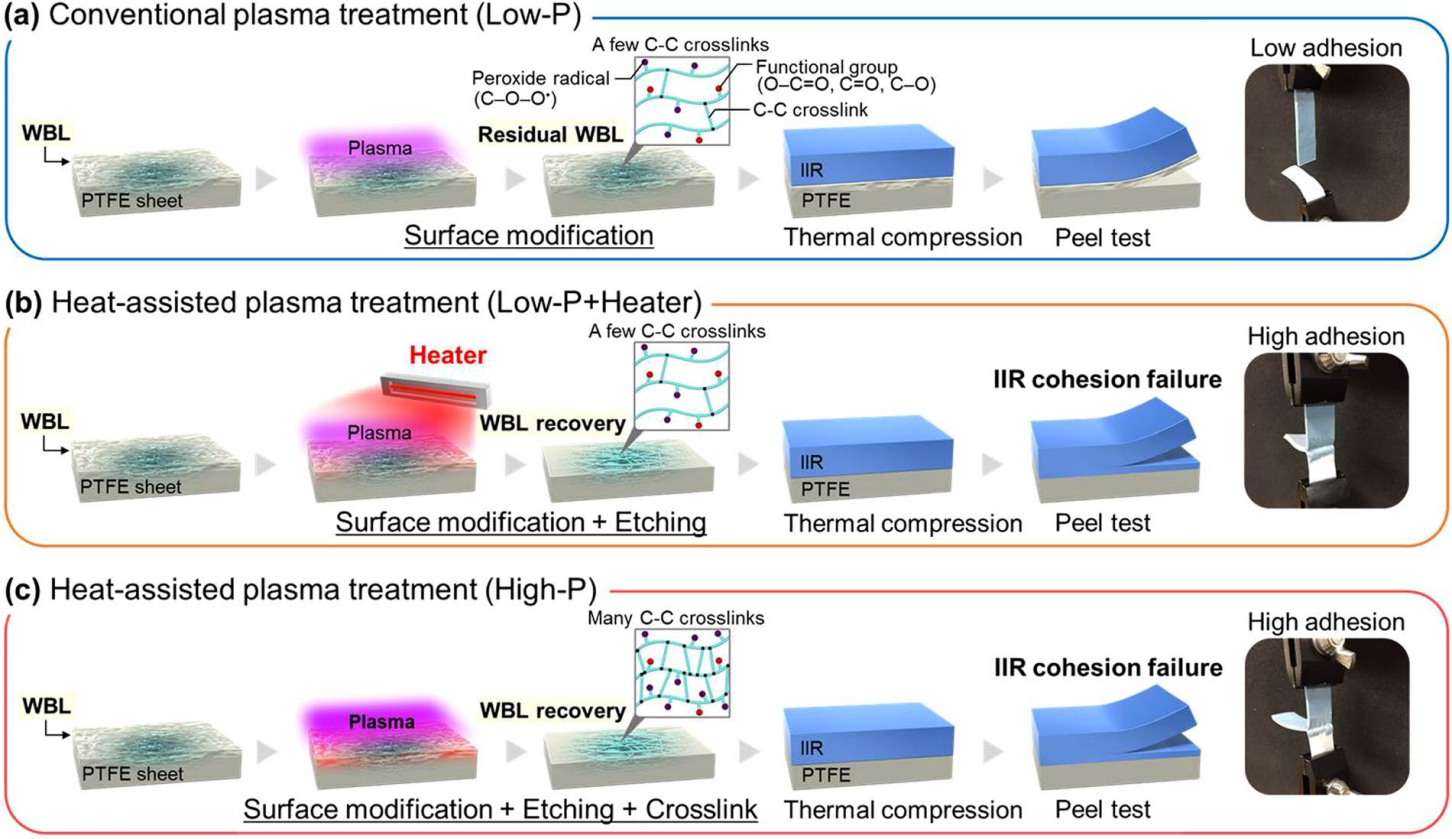
Model showing the adhesion improvement for the plasma-treated PTFE (**a**) Low-P; conventional plasma treatment, (**b**) Low-P+Heater; heat-assisted plasma treatment, and (**c**) High-P; heat-assisted plasma treatment.

We compared two types of heat-assisted plasma treatment procedure to improve the adhesion strength of PTFE surfaces, Low-P+Heater and High-P (see Table 1). The models proposed for the adhesion and peeling processes are shown in Fig. 8. Both heat-assisted plasma treatments drastically improved the adhesion property of PTFE, which resulted in IIR material failure in the middle of the T-peel test. However, the surface composition and the recovery of WBL differed. For the Low-P+Heater PTFE sample, etching was dominant. In contrast, both etching and the formation of C–C cross-links occurred for the High-P PTFE sample. In both cases, the recovery of WBL on the PTFE surface was the most important factor for improving the adhesion property. The heat-assisted plasma treatment using a heater is expected as an alternative method to dip treatment using sodium-containing solutions. Also, the heat-assisted plasma treatment could be applied not only to PTFE but also various to polymers.

## Methods

### Materials

Commercially available fluorinated resins (NITOFLON^®^No. 900UL, Nitto Denko, thickness: 0.2 mm) were cut into the size of 35 mm × 70 mm × 0.2 mm and used as PTFE specimens. Unvulcanized isobutylene–isoprene rubber (IIR) sheets (thickness ca. 2 mm) were prepared based on the patent procedure^23^.

### Sample preparation by plasma treatment

PTFE sheets were sequentially washed with acetone (99.5%, Kishida Chemical) and pure water for 1 min each using an ultrasonic bath (USK-1R, AS- ONE). The washed PTFE sheets were then dried using an air gun of N_2_ gas (99.99%, Neriki Gas). The dried PTFE sheets were then fixed on a cylindrical rotation stage. Finally, the PTFE sheets were plasma-treated using He gas (99.99%, Neriki Gas) under atmospheric pressure in a custom-made chamber system (Meisyo Kiko)^24^. Supplementary Information S-2 shows the schematic diagram of the custom-made chamber system added to a halogen line heater (LHW30, Fintech Tokyo). The surface temperature of the PTFE samples during plasma treatment was measured by using a digital radiation thermometer system (FT-H40K and FT-50A, Keyence) and controlled by using a switching power supply (PS5R-A24, Idec Izumi).

### Adhesion strength test

The adhesion properties of the plasma-treated PTFE were evaluated by measuring the adhesion strength of the IIR/PTFE assembly. Firstly, the plasma-treated PTFE samples were placed on the unvulcanized IIR sheets in the mold. Then, the assembly samples were compressed at almost 10 MPa at 180 °C for 10 min by using a hot-pressing machine (AH-2003, AS-ONE). Here, no adhesives were used in the adhesion process. Then, the sample temperatures of the IIR/PTFE assembly were returned to a room temperature. The adhesion strengths of the IIR/PTFE assembly were measured by a T-peel test using a digital force gage (ZP-200N, Imada) and an electric-driven stand (MX-500N, Imada). The sweep rate was 60 mm/min. The adhesion strengths were calculated by dividing the average tensile strength by the width of the IIR/PTFE assembly. Three samples were prepared under each of the same condition for confirming the reproducibility.

### Electron spin resonance measurements

To examine the radical density ratio of the plasma-treated PTFE, electron spin resonance (ESR) measurements were conducted by using a JES-FA100x (JEOL) with an X band. The plasma-treated PTFE sample was cut into 3 mm × 30 mm × 0.2 mm specimens and inserted into a quartz glass cell (inner diameter 3 mm). The microwave power and the applied frequency were set to 10 mW and 10 GHz, respectively. ESR spectra were obtained at room temperature in the range of 329 to 341 mT. The fourth signal (*g*_4_ = 1.981) of Mn^2+^ in MgO was used as a reference. The radical density ratios were calculated by double integration of the intensity attributed to peroxide radicals. Each radical density ratio was normalized to the ESR spectrum obtained at the lowest applied RF power density of 8.3 W/cm^2^.

### X-ray photoelectron spectroscopy

To examine the change of chemical components on the plasma-treated PTFE surface, angular-dependent X-rayphotoelectron spectroscopy (XPS) measurements were conducted by using a Quantum 2000 instrument (ULVAC-PHI) attached to an Al-*K*α source with take-off angles of 15, 45, and 75°. The area of X-ray irradiation was *Φ* = 100 μm, the pass energy was 23.50 eV, and the step size was 0.05 eV. The C1s-XPS spectra were collected between 280 and 296 eV. The cumulative numbers of the measurement were three. During the XPS measurement, the low-speed electron beam and the Ar ion beam were irradiated for the measured samples to neutralize the charges of them. The binding energies of as-received and plasma-treated PTFE were referenced to the peak indexed to −CF_2_− at 292.5 eV and 291.8 eV, respectively^20, 25^.

### Surface hardness test

To examine the surface hardness of the plasma-treated PTFE samples, load–depth data were collected from 0 to 40 μN with 20 ms intervals using an ENT-2100 (Elionix). The indentation hardness was calculated by dividing a maximum load by the projected contact area. The surface hardness distributions were obtained by measuring the indentation hardness at 50 different points for each PTFE sample. The average surface hardness of the plasma-treated PTFE samples was then obtained as a geometric mean.

### Surface topography

The surfaces of the plasma-treated PTFE samples were observed using a scanning electron microscope (SEM, JCM-6000, JEOL). Before observation, Au was vapor-deposited on the plasma-treated PTFE samples for preventing them from electrification. In addition, the surfaces were also observed in detail using a scanning probe microscope (SPM, SPM-9700, Shimadzu) with a micro cantilever (OMCL-AC160TN-R3, OLYMPUS) under atomic-force-microscope (AFM) tapping mode (cyclic contact mode).

## Electronic supplementary material

Dataset 1
