# Supplementary material for: Drastic Improvement in Adhesion Property of Polytetrafluoroethylene (PTFE) via Heat-Assisted Plasma Treatment Using a Heater
Source: Sci Rep. 2017 Aug 25;7:9476. doi: 10.1038/s41598-017-09901-y (PMC5573392; doi:10.1038/s41598-017-09901-y)

Supplementary Information

Drastic Improvement in Adhesion Property of Polytetrafluoroethylene (PTFE) via Heat-Assisted Plasma Treatment Using a Heater

Yuji Ohkubo,^1,*^ Kento Ishihara,^1^ Masafumi Shibahara,^2^ Asahiro Nagatani,^2^ Koji Honda,^2^ Katsuyoshi Endo^1^ and Kazuya Yamamura^1^

^1^Graduate School of Engineering, Osaka University, 2-1 Yamadaoka, Suita, Osaka 565-0871, Japan;

^2^Hyogo Prefectural Institute of Technology, 3-1-12 Yukihiracho, Kobe, Hyogo 654-0037, Japan

**Contents**

- Angular-dependent XPS results ………………………………………………… S-1
- Schematic diagram ……………………………………………………………… S-2

**S-1** Angular-dependent XPS results. (a) angular-dependent XPS spectra of Low-P, (b) angular-dependent XPS spectra of Low-P+Heater, (c) angular-dependent XPS spectra of High-P, and (d) ratio of functional groups calculated from peak resolution of the angular-dependent XPS spectra.

**
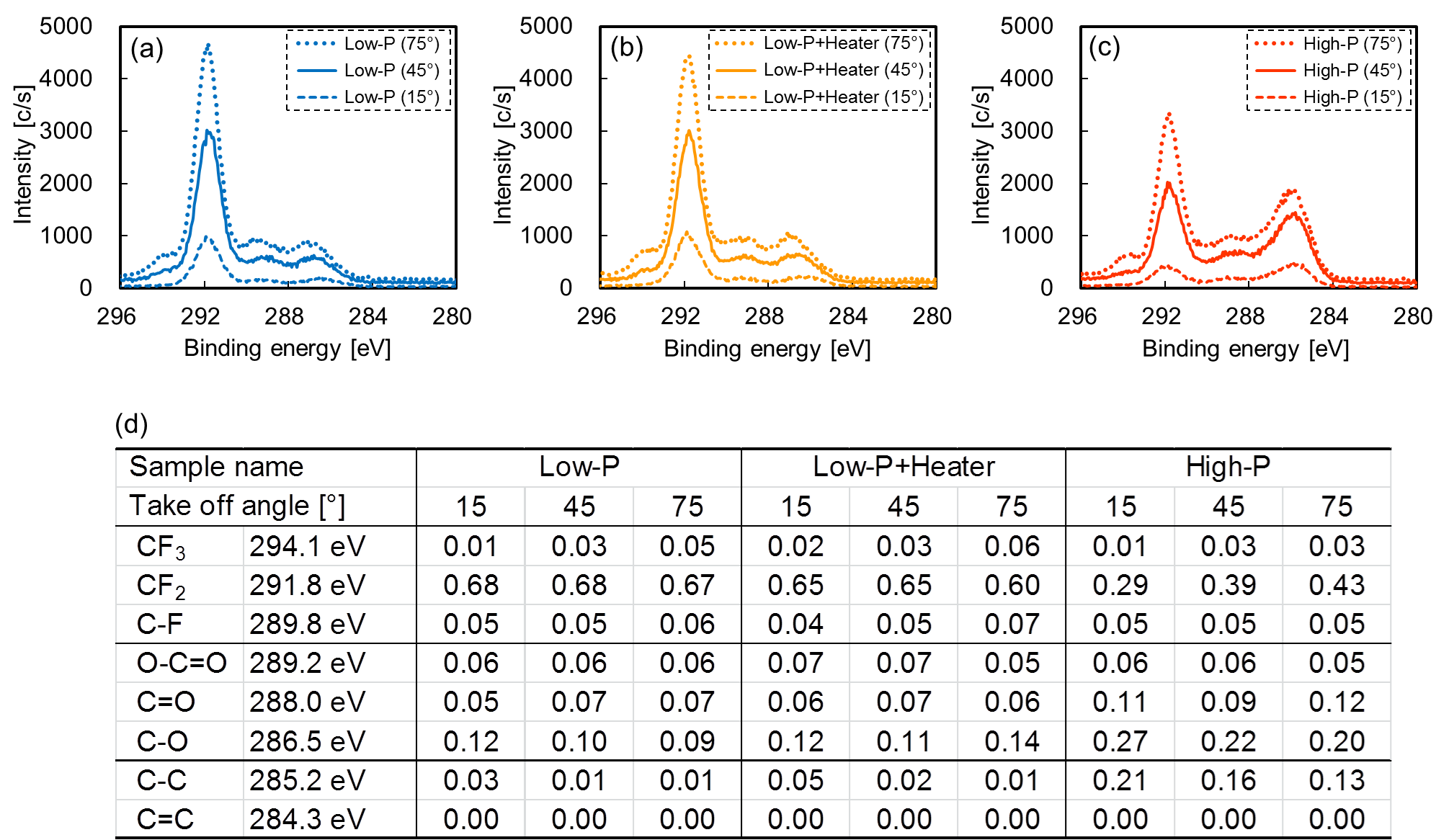
**

**S-2** Schematic diagram of the custom-made chamber system of the plasma processing equipment containing a halogen line heater. (a) front view and (b) side view.


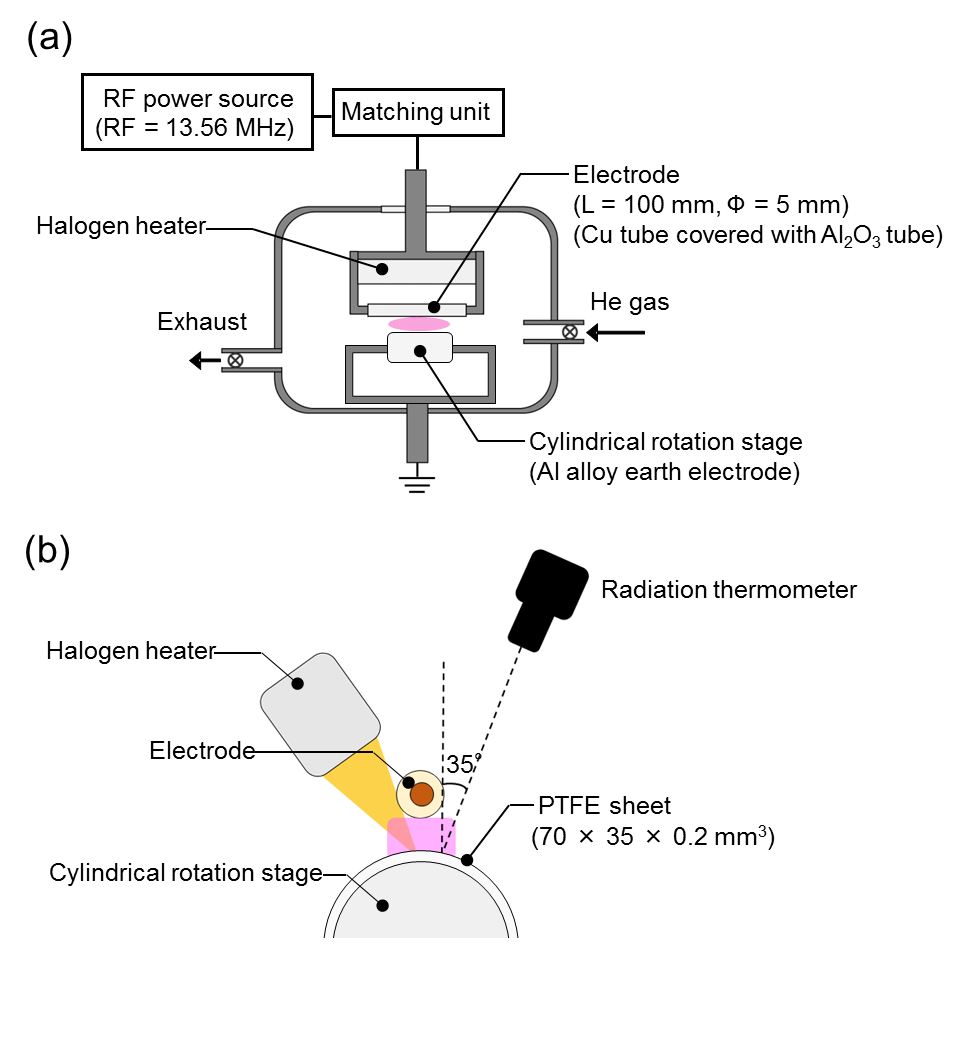

Supplement: Supplementary file 1 — Dataset 1 [file 41598_2017_9901_MOESM1_ESM.doc]
